# Books

**Published:** 1882-11

**Authors:** 


### Books.

MATERIA MEDICA AND THERAPEUTICS. INORGANIC SUBSTANCES. By Charles D. F. Phillips, M.D., M.R.C.P. Edited and adapted to the United States Pharmacopœia, by Lawrence Johnson, A.M., M.D. Vol. I and II. New York: William Wood & Co. 1882. Wood's Library of Standard Medical Authors.

This excellent work is intended to supplement and complete the treatise, by the same author, upon the Vegetable Kingdom, published three years ago. In these volumes he has devoted more space than is customary in modern text books to pharmaceutical chemistry; and the physiological action of drugs, in order to meet the modern popular demand, is accorded ample attention.

In the chapter on iodine we note the following practical suggestions: "In this distressing malady (hay-asthma) iodide of ammonium, combined with arsenic, will often give a better result than either remedy alone. Weak iodine solutions should be injected into the nares, or, what is more convenient, the vapor of iodine, or of carbolate of iodine, should be inhaled by the nostrils."

The chapters on Water, mineral waters and sea-bathing, are especially interesting and instructive.

The convenience and value of the work for reference is seriously impaired by the absence of an index appended to volume one. The index for the two volumes is consolidated and found alone at the end of the second volume.

In addition, however, to the ordinary index to the different inorganic medicinal substances treated of, we find, also, a good index—printed alone in volume two—to the various diseases in which the remedies are thought to be applicable.

We find grouped in these two volumes much important information not accessible to the practitioner in the more familiar works on materia medica.

---

ESSENTIALS OF VACCINATION; A Compilation of Facts Relating to Vaccine Inoculation and its Influence in the Prevention of Small-pox. By R. H. Hardaway, M.D. Chicago: Jansen, McClurg & Co. 1882. Pp. 146.

Considering the universality of the simple procedure known as vaccination, the ignorance which prevails, even among many well-qualified medical practitioners, concerning the details of the process and the problems involved in the theory of substitution which it typifies, is a constant source of astonishment to those who have made themselves familiar with the subject.

In this excellent little volume we find the more important facts relating to this topic succinctly, plainly and attractively set forth; the history of this transcendent discovery is briefly narrated, and the curious phases, as well as the practical points of the theme, are pleasingly presented.

"In 1830," says the writer, "Dr. Sonderland excited variola in cows by enveloping them in infected bed clothing. In a few days the animals manifested the usual symptoms of cow-pox, and the vesicles thus produced gave rise to genuine vaccine vesicles, when introduced into the human system. In 1836 Dr. Theile succeeded in inducing vaccinia in cows by variolation. The lymph thus generated was successfully used in vaccination, and many subsequently had their immunity tested by variolous inoculation and exposure to small-pox."

It has even been claimed that protective vaccinia can be produced in the human subject by the direct introduction of variolus virus diluted with warm milk, without the intervention of the cow. This theory is mentioned, but not commented upon by the writer.

"Finally," says he, "as is well known, the vaccine disease may be induced in the ox tribe by inoculation with virus derived directly from other cows laboring under the natural, casual or inoculated form of the disease; with the virus of horse-pox; with the virus which, originally derived from the cow or horse, has become humanized by passing through the human system, or, lastly, with the virus of human small-pox."

The pages of this valuable little book teem with useful knowledge respecting this all-important subject—knowledge which should be at the finger's end of every medical man, and we modestly submit that the surest and the best way to secure it is by a studious perusal of the volume which the learning and the labor of Prof. Hardaway, through his publishers, offer to the profession.

The binding, paper and print are all that could be desired.

---

Diseases of the Rectum and Anus. By Charles B. Kelsey, M.D. New York: William Wood & Co. 1882. Wood's Library of Standard Medical Authors.

The author claims that the advances which have been made within the last few years in this special branch of surgery have been very great. He thinks the present century will always be famous for the conspicuous advances which have been accomplished in abdominal surgery. New methods of treatment and new means of palliation in a large number of disorders have been proposed, and many have been generally adopted as trustworthy improvements. Numerous surgical expedients have, after critical tests, become recognized as legitimare procedures. These, and other improvements, have been achieved by workers all over the civilized world, and it has been his aim to collect and condense within the pages of this volume what is positively known, and to present it to his readers in a convenient and acceptable shape.

In the chapter on the treatment of internal hemorrhoids, we notice the injection of carbolic acid very favorably mentioned. Care should be observed to avoid using a solution of too great strength. One part of the acid to six each of glycerine and water is recommended as effective, and yet not liable to cause ulceration and sloughing. Of this solution five drops is the proper amount to use.

In speaking of this procedure he says: "Although I should be slow to advocate any one treatment of this affection to the exclusion of all others, I now often adopt this where Allingham's operation (the ligature) is declined by the patient, and as yet I have not known it to fail. Its advantages over all other methods, provided its results prove equally satisfactory, are manifest to all. The patient is not terrified at the outset by the prospect of a surgical operation, is not confined to his bed, and is not subjected to any suffering. The cure goes on painlessly and almost without his consciousness."

It must sometimes, however, be a very tedious, even if a very successful means of relief, for on the next page we read: "Each injection should be followed by a day's rest in the horizontal position." "Only one tumor should be injected at a time and I seldom repeat the injections oftener than once a week. It will sometimes be found necessary to inject the same tumor twice or three times when it is a very large one."

Of course the operation by the ligature is unreservedly commended. Of this mode of treatment he says: "It is as safe as any operation in surgery, and by it the surgeon may promise his patient an absolute and permanent cure of his troubles in every case. This is saying a great deal, but not too much."

The reader cannot be otherwise than favorably impressed by this practical treatise on an important and interesting subject.

---

INDEX-CATALOGUE OF THE LIBRARY OF THE SURGEON-GENERALS OFFICE, UNITED STATES ARMY. Authors and Subjects. Volume iii. Chclecynanin-Dzondi. Washington: Government Printing Office. 1882. Pp. 1020.

This monument of patient and laborious application is the third of the whole series of ten volumes to be issued. They have heretofore appeared at the rate of one volume a year. Volume third contains 9,043 author-titles, representing 10,076 volumes and 7,386 pamphlets. It also includes 8,572 subject-titles of separate books and pamphlets, and 28,846 titles of articles in periodicals. There are also catalogued 4,335 medical portraits.

To convey a faint idea of the magnitude of this work it will only be necessary to state that 151 of the royal octavo, finely printed pages, are monopolized by "cholera" alone.

A limited number of copies can be procured, upon application to the government printer, at the actual cost of material and press work, plus ten per cent. The total cost will amount to about $2.00 per volume.

---

THE PHYSCIAN HIMSELF AND WHAT HE SHOULD ADD TO HIS SCIENTIFIC ACQUIREMENTS. By D. W. Cathell, M.D. Second Edition, Carefully Revised. Baltimore: Cushings & Bailey. 1882. Pp. 208. Price, $1.25.

It is not often that the medical journalist is called upon to note the appearance of the second edition of a work so soon after its first advent as it is our pleasure to do in the present instance.

The rapid exhaustion of the first edition of Dr. Cathell's unique production is the best evidence the author could have of the appreciation in which his work is held. It is undoubtedly a *taking* book and, in the main, deserves, we think, the cordial reception which has been extended to it.

The second edition is an improvement, in some respects, upon the first. The contents have been divided into chapters and the rearrangement of various paragraphs is advantageous.

---

Transactions of the Mississippi State Medical Association at the Fifteenth Annual Session, Held at Oxford April 5–7, 1882. Wirt Jonston, M.D., Jackson, President. T. W. Fullilove, M.D., Vaiden, Secretary.

This is a handsome paper-covered volume of 170 pages, containing besides the proceedings of the annual session, the presidents address on Alcohol, twelve papers by contributors on various subjects and a comprehensive report on surgery prepared by M. S. Craft, M.D., and composed of sub-reports from eighteen callaborators residing in different portions of the State.

Among the voluntary reports we note a paper on Traumatic tetanus, by Dr. H. A. Grant; Abortive treatment of puerperal convulsions, by Dr. T. H. Gordon; Puerperal eclampsia successfully treated with veratrum viride, by Dr. N. L. Guice; Case of puforation of the ilium by worms, by Dr. T. J. Cofford and Spinal curvature, by Dr. Chesley Daniel.

The typographical appearance of the book would be greatly improved by the use of uniform type for the captions of the different papers.

---

The Physicians' Visiting List for 1883. Philadelphia: P. Blakiston, Son & Co.

For thirty years this house has issued these useful aids to the practitioner's memory. The volume before us contains an almanac, table of signs, Marshall Hall's ready method in asphyxia, list of poisons and antidotes, the metric and French systems of weights and measures, posological table and table for calculating the period of utero-gestation. Each volume is supplied with a tuck, pocket and pencil, and the prices range from $1 00 for twenty-five patients weekly to $3.00 for the interleaved edition arranged for fifty patients. It is complete, compact and convenient.

